# Benefits of intracorporeal gastrointestinal anastomosis following laparoscopic distal gastrectomy

**DOI:** 10.1186/1477-7819-10-267

**Published:** 2012-12-12

**Authors:** Sang-Woong Lee, Nobuhiko Tanigawa, Eiji Nomura, Takaya Tokuhara, Masaru Kawai, Kazutake Yokoyama, Masako Hiramatsu, Junji Okuda, Kazuhisa Uchiyama

**Affiliations:** 1 Department of General and Gastroenterological Surgery, Osaka Medical College, 2-7, Daigaku-Machi, Takatsuki City Osaka 569-8686, Japan; 2 Tanigawa Memorial Hospital, Kasuga 1-Chome, Ibaraki City, Osaka 567-0031, Japan

**Keywords:** Laparoscopic distal gastrectomy, Intracorporeal anastomosis, Extracorporeal anastomosis, Billroth I, Roux-en-Y

## Abstract

**Background:**

Laparoscopic gastrectomy has recently been gaining popularity as a treatment for cancer; however, little is known about the benefits of intracorporeal (IC) gastrointestinal anastomosis with pure laparoscopic distal gastrectomy (LDG) compared with extracorporeal (EC) anastomosis with laparoscopy-assisted distal gastrectomy (LADG).

**Methods:**

Between June 2000 and December 2011, we assessed 449 consecutive patients with early-stage gastric cancer who underwent LDG. The patients were classified into three groups according to the method of reconstruction LADG followed by EC hand-sewn anastomosis (LADG + EC) (n = 73), using any of three anastomosis methods (Billroth-I (B-I), Billroth-II (B-II) or Roux-en-Y (R-Y); LDG followed by IC B-I anastomosis (LDG + B-I) (n = 248); or LDG followed by IC R-Y anastomosis (LDG + R-Y) (n = 128)). The analyzed parameters included patient and tumor characteristics, operation details, and post-operative outcomes.

**Results:**

The tumor location was significantly more proximal in the LDG + R-Y group than in the LDG + B-I group (*P* < 0.01). Mean operation time, intra-operative blood loss, and the length of post-operative hospital stay were all shortest in the LDG + B-I group (*P* < 0.05). Regarding post-operative morbidities, anastomosis-related complications occurred significantly less frequently in with the LDG + B-I group than in the LADG + EC group (*P* < 0.01), whereas there were no differences in the other parameters of patients’ characteristics.

**Conclusions:**

Intracorporeal mechanical anastomosis by either the B-I or R-Y method following LDG has several advantages over at the LADG + EC, including small wound size, reduced invasiveness, and safe anastomosis. Although additional randomized control studies are warranted to confirm these findings, we consider that pure LDG is a useful technique for patients with early gastric cancer.

## Background

Since the technique of laparoscopy-assisted Billroth-I gastrectomy was first reported by Kitano and colleagues in 1994 [1], laparoscopic gastrectomy for cancer (LGC) has been gaining increasing popularity worldwide because it is associated with earlier patient recovery compared with open surgery [2-4]. A national survey conducted by the Japan Society of Endoscopic Surgery (JSES) every 2 years has shown increasing use of laparoscopic procedures for gastric cancer in Japan [5]. According to the 10th JSES survey, more than 7,300 patients underwent LGC in 2009, which equated to 25.9% of 28,600 patients with gastric cancer who underwent open gastrectomy, LGC or endoscopic treatment such as endoscopic mucosal resection or and endoscopic sub-mucosal resection in the same institutions. Laparoscopic distal gastrectomy (LDG) was the most commonly performed type of LGC (75.7% of operation). The survey also reported the incidence of post-operative complications in a total of 10,355 LDGs performed in 2008 and 2009 [6], with the most frequent being stomal stenosis (2.0%), followed by pancreatitis or pancreatic fistula formation (1.3%), anastomotic leakage (1.1%), wound infection, peritoneal abscess, bleeding, pneumonia, and ileus. This suggests that anastomosis-related complications are the most common complication subsequent to LDG. In the context of gastrointestinal reconstruction, the majority of anastomoses following LDG were performed by laparoscopy-assisted procedures through a mini-laparotomy incision of 60 to 70 mm in length made on the epigastrium. In such a laparoscopy-assisted distal gastrectomy (LADG) [1] procedure, gastrointestinal reconstructive anastomosis is extracorporeally conducted in a limited working space with restricted vision, and it is possible that this may lead to increased risk of anastomotic leakage.

Although we originally began performing LADG, in the hope of overcoming the drawbacks of cumbersome reconstruction, we introduced intracorporeal (IC) stapled gastroduodenostomy and gastrojejunostomy in association with LDG in 2004 [7,8]. We then developed two methods for IC reconstructive anastomosis following LDG: 1) IC Billroth I anastomosis (B-I) was used when there was no tension expected at the gastroduodenal anastomosis, and 2) IC Roux-en-Y anastomosis (R-Y) was used when there were some concerns about strain.

We report our experience and results of three kinds of gastrointestinal reconstructive anastomosis: LADG followed by extracorporeal hand-sewn anastomosis (LADG + EC), LDG + B-I , and LDG + R-Y.

## Methods

Patients who had undergone LDG for gastric cancer during the period June 2000 to December 2011 in the Department of General and Gastroenterological Surgery, Osaka Medical College, Japan, were assessed in terms of their clinical outcomes subsequent to gastrointestinal reconstructive anastomosis following surgery.

### Indications for laparoscopic gastrectomy in cancer

Indications for LGC at our institute include all tumors confined to the muscularis propria that are not amenable to endoscopic mucosal resection, with lymph-node involvement limited to N1. Patients requiring salvage surgery after incomplete endoscopic resection are also included. Operations were converted to open surgeries when serosal invasion or extensive lymphadenopathy was detected during laparoscopy. LDG is indicated for distal and middle third gastric cancers in which tumor margins of at least 20 mm for early and 30 to 50 mm for advanced lesions are possible. Some patients with very early disease may have a more limited resection such as pylorus-preserving or segmental gastrectomy [9,10].

### Surgical techniques

The LDG procedure was carried out in all cases as follows. A 12-mm trocar was inserted through an umbilical wound, and pneumoperitoneum was established. Another 12-mm trocar was inserted 10 mm above and to the left of the umbilicus, and a 5-mm trocar was inserted 20 to 30 mm above and to the right of the umbilicus under laparoscopic guidance. A 5-mm trocar was inserted into each of the right and left costal margins, respectively. The intra-abdominal pressure was maintained at a constant 8 to 12 mmHg. After inspection of the peritoneal cavity, mobilization of the stomach and dissection of the lymph nodes were carried out as described previously [8,11,12].

Surgical procedures for gastrointestinal reconstructive anastomosis after LDG were as follows. For patients operated on before May 2004, LDG + EC was generally used, and for subsequent operations, one of the two IC mechanical anastomosis techniques were performed after LDG. Of the two IC methods, B-I reconstruction was the choice of reconstruction for LDG where possible, however, R-Y reconstruction was used when tension was expected on the anastomosis, when the patient had reflux esophagitis or a hiatus hernia, or when the patient was elderly and/or high risk [11].

#### Extracorporeal anastomosis in laparoscopy-assisted distal gastrectomy

The mini-laparotomy wound 60 to 70 mm long was made in the upper midline and opened to allow insertion of a wound protector. An end-to-end gastroduodenostomy (B-I) or end-to-side gastrojejunostomy (Billroth II (B-II) or R-Y) was created to allow open surgery between the gastric stump and duodenal stump or the biliopancreatic jejunum, using a two-layer hand-sewn anastomosis technique.

#### Intracorporeal anastomosis in pure laparoscopic distal gastrectomy

##### Billroth I gastroduodenostomy

**Figure 1:**
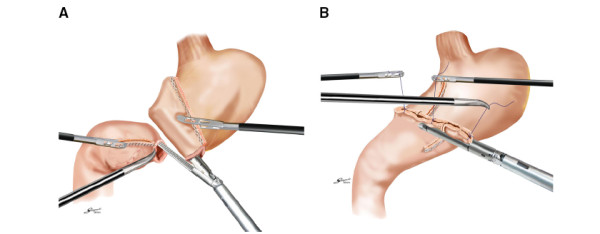
**Intracorporeal Billroth I anastomosis.** (**A**) A side-to-side gastroduodenostomy was formed by firing the 45-mm linear stapler; and (**B**) the common enterotomy was closed with two further firings of the stapler.

The original method for this procedure was first described by Kanaya *et al*. [7]. We partly modified the technique for ease of use, as follows. A gastrotomy was performed on the greater curve corner of the staple line on the remnant stomach, then a small hole was made on the posterior tip of the duodenal stump. The cartridge fork of the 45-mm linear stapler was inserted into the gastric remnant, and another fork was inserted into the duodenal stump. This was followed by firing of the stapler to form the functional end-to-end gastroduodenal anastomosis. After the anastomosis was inspected from the lumen to check for bleeding, the common enterotomy was apposed vertically with three intracorporeally placed stay sutures, and closed with two further firings of the stapler. This strategy resulted in a satisfactory V-shaped anastomosis, and security was confirmed with an air-leak test at the end of the anastomosis (Figure 1A, B).

##### Roux-en-Y reconstruction

**Figure 2:**
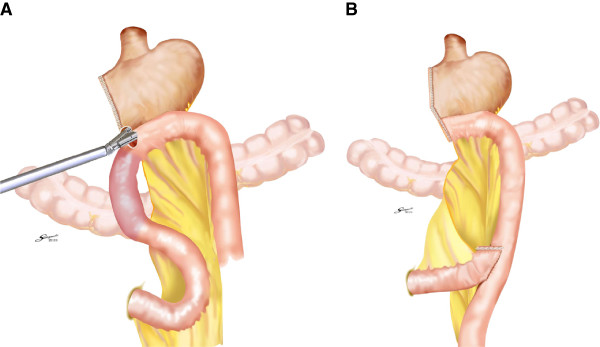
**Intracorporeal Roux**-**en**-**Y anastomosis.** (**A**) A side-to-side gastrojejunostomy was performed by firing a 60-mm linear stapler, then the remaining enterotomy was closed with a further firing of the linear stapler. (**B**) An end-to-side jejunojejunostomy was created between the descending alimentary jejunum and the biliopancreatic jejunum, using a linear stapler and sutures.

We have previously published descriptions of this method [8,12]. In brief, a distance of 200 mm was measured on the jejunum from the ligament of Treitz, and a sacrifice jejunum was created 70 mm distally by division of the corresponding jejunal mesentery. Following this maneuver, the sacrifice jejunum became discolored as a result of ischemia. A small enterotomy was made on the healthy part of the bowel just distal to the sacrifice jejunum. The cartridge fork of the 60-mm linear stapler was inserted into the enterotomy on the jejunum, and the other fork of the linear stapler was then inserted into the stomach through a gastrostomy made on the greater curve corner of the gastric stump. A side-to-side gastrojejunostomy was formed by firing the stapler. The anastomosis was checked from the lumen for any bleeding, which was controlled by bipolar coagulation. The remaining enterotomy was closed with a further firing of the linear stapler. The jejunum was divided simultaneously to complete the anti-peristaltic side-to-side gastrojejunostomy (Figure 2A). Finally, using a linear stapler and laparoscopic sutures, a side-to-side jejunojejunostomy was fashioned between the descending alimentary jejunum and the biliopancreatic jejunum (Figure 2B).

#### Definition of post-operative complications

The diagnosis of a clinically relevant anastomotic leak was based on clinical signs, including an inflammatory reaction requiring treatment and confirmation of the insufficiency, either endoscopically or radiologically by computed tomography or using a contrast medium (diatrizoate meglumine and diatrizoate sodium; Gastrografin; Bracco Diagnostics Inc., Princeton, NJ, USA). Anastomotic stenosis was defined as any form of narrowing in the anastomosis region by contrast swallow studies or gastroscopy (≤10 mm in diameter) and any symptom of dysphagia when swallowing solid, semi-solid or liquid nourishment, which then required, endoscopic dilation. Gastric stasis was defined if the patients exhibited symptoms such as upper abdominal distension and remnant stomach fullness on X-ray, or if the patient required starvation for longer than 24 hours.

Pancreatic fistula (PF) was defined by the International Study Group Pancreatic Fistula (ISGPF) criteria [13], and divided into four categories: no PF, biochemical PF without clinical sequelae (grade A), PF requiring any therapeutic intervention (grade B), and PF with severe clinical sequelae (grade C). Clinically relevant PF was defined as grades B and C, in accordance with the ISGPF grading system.

#### Statistical analysis

Statistical analysis of the data was performed using SSPS (12.0; SPSS, Chicago, IL, USA). All data are presented as the mean ± standard deviation (SD) or as the number and percentage of patients. Continual variables are expressed as mean ± SD, and comparisons between groups were performed using the *t*-test and the Mann–Whitney *U*-test. Comparisons of categorical variables were performed by means of the Fisher exact test. *P* < 0.05 was considered significant.

## Results

LGC was performed on 769 patients: 449 (58.4%) underwent LADG or pure LDG, while the remainder consisted of 160 (20.8%) laparoscopic pylorus-preserving gastrectomies, 48 (6.2%) laparoscopic proximal gastrectomies, 47 (6.1%) laparoscopic segmental gastrectomies, 39 (5.1%) laparoscopic total gastrectomies, and 26 (3.4%) laparoscopic wedge resections. In the group of 449 patients with LADG or pure LDG, 73 had LADG with EC anastomosis, and 376 had pure LDG with IC anastomosis (248 B-I and 128 R-Y). The methods of EC anastomosis for the 73 LADG operations were 56 B-I, 9 R-Y, and 8 B-II.

### Patients’ characteristics of each reconstruction group

There were no significant differences in age, gender distribution, or body mass index (BMI) between the three reconstruction-method groups (Table 1). The mean age (mid-sixties), male:female ratio (around 15) and BMI (22 ± 3.1) were almost comparable for the three groups. The tumor locations were defined using the Japanese Classification of Gastric Carcinoma [14] as being in the upper (U), middle (M) or lower (L) third of the stomach. Regarding both IC techniques, there were 0 U, 121 M and 127 L in the LDG + B-I group, and 20 U, 89 M and 19 L in the LDG + R-Y group; that is, there were significantly more tumors located distally in the LDG + B-I than in the LDG + R-Y group (*P* < 0.01). However, for LADG, there was no significant difference in tumor location between the B-I (n = 56) and R-Y (n = 9) subgroups, probably because the number of operative cases of R-Y reconstruction in the LADG group was too small.

**Table 1:** Patient’s characteristics for three kinds of reconstruction

	**LADG + EC (n = 73)**^**a**^			**LDG + B-I (n = 248)**^**b**^	**LDG + R-Y (n = 128)**^**c**^
Age, years	64 ± 11			65 ± 11	66 ± 10
Male : female	48 : 25			147 : 101	90 : 38
BMI, kg/m^2^	22.3 ± 3.2			22.0 ± 3.1	22.6 ± 3.0
	Type of anastomosis				
	B-I	R-Y	B-II		
Tumor location					
Upper	0	1	2	0	20
Middle	24	4	6	121	89
Lower	32	4	0	127	19

Abbreviations: *BMI* body mass index; *B-I* Billroth I, *B-II* Billroth-II; *EC* extracorporeal, *IC* intracorporeal; *LADG* laparoscopy-assisted distal gastrectomy; *LDG* laparoscopic distal gastrectomy.

^a^LADG with any of the three types of EC anastomosis.

^b^Pure LDG with IC B-I.

^c^Pure LDG with IC R-Y.

### Intraoperative variables

The mean operating time was 261 ± 63 minutes for the LDG + B-I group, 333 ± 79 minutes for the LDG + R-Y group, and 339 ± 83 minutes for the LADG + EC group, respectively (Table 2). Operating time was significantly shorter for the LDG + B-I group than for the LDG + RY and LADG + EC groups (*P* < 0.01), whereas there was no significant difference in operating time between these latter two groups.

**Table 2:** Intraoperative variables for three groups

	**LADG + EC (n = 73)**^**a**^	**LDG + B-I (n = 248)**^**b**^	**LDG + R-Y (n = 128)**^**c**^
Operation time, min	339 ± 83^d^	261 ± 63^e^	333 ± 79^d^
Blood loss, ml	119 ± 108	47 ± 48^e^	76 ± 80^f^

Abbreviations: *B-I* Billroth I anastomosis; *B-II* Billroth II anastomosis; *EC* extracorporeal; *IC* intracorporeal; *LADG* laparoscopy-assisted distal gastrectomy; *LDG* laparoscopic distal gastrectomy; *R-Y* Roux-en Y anastomosis.

^a^LADG with any of the three types of anastomosis.

^b^Pure LDG with IC B-I.

^c^Pure LDG with IC R-Y.

^d^Groups were not significantly different from each other.

^e^*P* < 0.01 compared with the other two groups.

^f^*P* < 0.05 compared with the LADG group.

The intraoperative blood loss was 47 ± 48 ml for the LDG + B-I group, 76 ± 80 ml for the LDG + R-Y group, and 119 ± 108 ml for the LADG + EC group. Blood loss was significantly less for the LDG + B-I group than for the other two groups (*P* < 0.01), and it was also significantly less for the LDG + R-Y group than for the LADG + EC group (*P* < 0.05).

### Post-operative morbidity

Anastomotic leakage rates were 1.2% (3/248) for the LAD + B-I group, 1.6% (2/128) for the LAD + R-Y group, and 6.8% (5/73) for the LADG + EC group (7.1% (4/56) for B-I, 11.1% (1/9) for R-Y group with LADG and none (0/8) for B-II) (Table 3). Three of these patients required reoperation, and seven patients had collections that were treated by percutaneous drainage.

**Table 3:** Post-operative complications for three groups

	**LADG + EC (n = 73)**^**a**^	**LDG + B-I (n = 248)**^**b**^	**LDG + R-Y (n = 128)**^**c**^	***p *Value**
Anastomotic leakage,%^d^	5 (6.8)	3 (1.2)	2 (1.6)	0.04
Anastomotic stenosis,%	2 (2.7)	1 (0.4)	0	0.32
Gastric stasis,%	5(6.8)	3 (1.2)	3 (2.3)	0.06
Pancreatic fistula,%	2 (2.7)	12 (4.8)	2 (1.6)	0.40

Abbreviations: *B-I* Billroth I; *B-II* Billroth II; *LADG* laparoscopy-assisted distal gastrectomy; *LDG* laparoscopic distal gastrectomy; *R-Y* Roux-en Y anastomosis.

^a^LADG with any of the three types of anastomosis.

^b^Pure LDG with IC B-I.

^c^Pure LDG with IC R-Y.

^d^Bleeding from the anastomosis was not encountered in any of the patients.

Anastomotic stenosis was encountered in 0.4% (1/248) of the LDG + B-I group, none (0/128) of the LDG + R-Y group and 2.7% (2/73) of the LADG group (3.6% (2/56) for B-I, and 0% (0/17) for the other reconstruction types). All of the anastomotic strictures were successfully treated by endoscopic balloon dilatation.

Gastric stasis occurred in 1.2% (3/248) of the LDG + B-I group and in 2.3% (3/128) of the LDG + R-Y group, but was much higher at 6.8% (5/73) in the LADG + EC group (7.1% (4/56) for the B-I subgroup, 11.1% (1/9) for the R-Y subgroup and none for the B-II subgroup). Each case with gastric stasis improved spontaneously in 1 to 2 weeks.

Formation of PF (grades B and C of the ISGPF grading system) was found in 4.8% (12/248) of the LDG + B-I group, 1.6% (2/128) of the LDG + R-Y group and 2.7% (2/73) of the LADG + EC group. Although two of these patients had post-operative hemorrhage associated with pancreatic inflammation and secondary pseudoaneurysm of the gastroduodenal artery, both cases were successfully treated by endovascular coiling.

The length of post-operative hospital stay was significantly shorter for the LDG + B-I group (11.2 ± 0.7 days) than for the LDG + R-Y (17 ± 1.3 days) or LADG + EC (21.6 ± 11.2 days) groups (*P* < 0.05), whereas there was no significant difference between the latter two groups. This trend was similar to those for the operating time and intra-operative blood loss, suggesting that the surgical stresses represented by those parameters may be closely associated with the length of post-operative hospital stay.

## Discussion

Laparoscopic surgery is being been increasingly used for the treatment of gastric cancer over the past two decades, especially in East Asian countries such as Japan, Korea, China ,and Taiwan. In general, LGC can be divided into laparoscopy-assisted and pure laparoscopic techniques. With laparoscopy-assisted gastrectomy, lymph-node dissection is performed laparoscopically, but the transection of the stomach and the anastomosis are performed thorough an epigastric mini-laparotomy. Performing the anastomosis in this narrow and restricted space is often difficult, especially on obese patients with thick abdominal walls or on patients with a small remnant stomach.

Although we initially began performing LADG in the hope of overcoming the drawbacks of cumbersome reconstruction, we introduced the use of LDG followed by IC stapled gastroduodenostomy and gastrojejunostomy in 2004 [11]. To investigate the feasibility and benefits of IC anastomosis, we compared our experience of using LADG and pure LDG in the current study, using a consecutive series of patients in our institution. We found that the rates for anastomotic complications, including leakage, stenosis, and gastric stasis, were significantly lower for either method of IC mechanical anastomosis (B-I or R-Y) after LDG than for LADG follwed by hand-sewn anastomosis (using any of the three methods). The rate of anastomotic leakage in the IC groups (1.3%) seems to be within the permissible level compared with other accounts using these techniques [15,16]. Similarly, the rate of of 6.8% for anastomotic leakage after LADG (7.1% for B-I, 11.1% for R-Y and 0% for B-II ) in the current study was comparable with other reports using LADG, with a rate of 7.8% (8.1% (7/87) for B-I and 0% (0/3) for R-Y) reported by Fujiwara *et al*. [17] and 5.3% (4/76 for B-I with no case for R-Y reconstruction) by Shimizu *et al*. [18]. The relatively high incidences of anastomotic leakage were possibly associated with the results obtained from initial experience of LADG in each institution.

In the current study, the LADG + EC group had the largest blood loss and longest operating time of the three groups tested. Because patients underwent LADG until May 2004, when we changed our strategy to pure LDG plus IC anastomosis [8,11], our LADG results might have been subject to some degree of learning-curve effect while we gained experience of laparoscopy-assisted surgical techniques. However, although this dataset does therefore have the drawback of different time periods when each surgical procedure was performed, the reduced blood loss seen with pure LDG may be also reflective of the small wound length required and avoidance of cumbersome anastomosis through a minilaparotomy.

The current study also indicates that pure LDG + B-I resulted in a significantly smaller volume of blood loss and shorter operating time than did pure LDG + R-Y. The consequent reduction in surgical stresses, including operating time and blood loss, and the lower incidence of post-operative complications seemed to be associated with the fact that the LDG + B-I group also had the shortest length of post-operative hospital stay. It should be noted that the mean length of hospital stay in the current study was rather longer than reported elsewhere [19]; however, because the Japanese medical insurance system is structured differently from those in other countries, it is difficult to estimate the correct length of hospital stay based solely on surgical aspects.

In our institution, B-I reconstruction using the delta-shape method with linear staplers [7] was our first choice of reconstruction after LDG, with R-Y reconstruction (also with linear staplers) [8,12] reserved for selected cases, including those for which tension would be expected on the reconstruction, those with reflux esophagitis or a hiatus hernia, and those with elderly or high-risk patients. As indicated by the numbers of each reconstruction method used (248 B-I reconstructions and 128 R-Y reconstructions in the current consecutive series), B-I was used for the majority of patients who underwent distal gastrectomy.

With regard to post-operative nutritional status, we already confirmed that decreases in body weight and food intake at 12 months post-operatively compared with pre-operative values were significantly less for the LDG + B-I group than for the LDG + R-Y group. In addition, there were fewer subjective reports from patients about their small stomach in the LDG + B-I group than in the LDG + R-Y group [20]. Certainly, bile reflux into the remnant stomach and lower esophagus is one of the drawbacks for B-I reconstruction compared with R-Y reconstruction. It may be closely associated with the development of remnant gastritis, mainly caused by bile reflux [21]. However, as we recently suggested, based on another nationwide survey, the risk of the development of remnant gastric cancer does not appear to be directly associated with the reconstruction method [22].

The development of our strategy for digestive reconstruction after LDG is indicative of the trend toward IC mechanical reconstruction, which offers advantages in wound length and avoidance of tension through a mini-laparotomy during cumbersome anastomosis. For LDG + R-Y reconstruction, we were able to successfully use IC jejunojejunostomy, which saved a further 20 mm length for the umbilical wound [12]. This also allowed for reduced manipulation of the bowel, and was particularly useful for obese patients, for whom access through a mini-laparotomy can be limited. Reconstruction was performed under continuous laparoscopic guidance, and the disorientating and time-consuming switch to open surgery was thus avoided. In addition, totally IC laparoscopic gastrectomy has been shown to lead to earlier bowel function recovery compared with laparoscopy-assisted and open resections [23]. As indicated by others [15,23], IC anastomosis is more costly than EC anastomosis, because it requires three to four applications of endoscopic linear stapler cartridges for the anastomosis. We are currently trying to lower the cost by closing the entry hole using an IC hand-sewn technique instead of stapling [24].

## Conclusions

In conclusion, we found in this study that LDG followed by IC mechanical anastomosis using either the B-I or R-Y method has several advantages over LADG followed by EC hand-sewn anastomosis, including small wound size, reduced invasiveness, and more effective anastomosis. Although additional randomized control studies are warranted to confirm these findings, we consider that pure LDG is a useful technique for patients with early gastric cancer.

## Competing interests

None of the authors have any conflicts of interest or financial ties to disclose.

## Authors’ contributions

SL designed and conducted the study, analyzed the data, and helped to write the manuscript. NT helped to design the study, conducted surgical operations, and helped to write the manuscript. EN conducted surgical operations and helped to write the manuscript. TT, MK, KY, MH, and JO helped to design the study and helped to write the manuscript. KU is the principal investigator, and designed the study, assisted in writing, revising and editing the manuscript. All authors approved the final manuscript.
